# Development and Long-Term Stability of a Novel Microbial Fuel Cell BOD Sensor with MnO_2_ Catalyst

**DOI:** 10.3390/ijms18020276

**Published:** 2017-01-28

**Authors:** Shailesh Kharkwal, Yi Chao Tan, Min Lu, How Yong Ng

**Affiliations:** 1 Centre for Water Research, Department of Civil & Environmental Engineering, National University of Singapore, Engineering Drive 3, Singapore 117580, Singapore; yichao@u.nus.edu; 2 Key Laboratory of Flexible Electronics (KLOFE), Institute of Advanced Materials (IAM), Jiangsu National Synergetic Innovation Center for Advanced Materials (SICAM), Nanjing Tech University (NanjingTech), Nanjing 211816, China; iammlv@njtech.edu.cn

**Keywords:** microbial fuel cell, manganese dioxide, cathode, biochemical oxygen demand, biosensor, wastewater

## Abstract

A novel microbial fuel cell (MFC)-based biosensor was designed for continuous monitoring of biochemical oxygen demand (BOD) in real wastewater. To lower the material cost, manganese dioxide (MnO_2_) was tested as an innovative cathode catalyst for oxygen reduction in a single chamber air-cathode MFC, and two different crystalline structures obtained during synthesis of MnO_2_ (namely β- and γ-MnO_2_) were compared. The BOD sensor was studied in a comprehensive way, using both sodium acetate solution and real domestic wastewater (DWW). The optimal performance of the sensor was obtained with a β-MnO_2_ catalyst, with *R*^2^ values of 0.99 and 0.98 using sodium acetate solution and DWW, respectively. The BOD values predicted by the β-MnO_2_ biosensor for DWW were in agreement with the BOD_5_ values, determined according to standard methods, with slight variations in the range from 3% to 12%. Finally, the long-term stability of the BOD biosensor was evaluated over 1.5 years. To the best of our knowledge, this is the first report of an MFC BOD sensor using an MnO_2_ catalyst at the cathode; the feasibility of using a low-cost catalyst in an MFC for online measurement of BOD in real wastewater broadens the scope of applications for such devices.

## 1. Introduction

To ensure efficient operation of wastewater treatment processes, the continuous monitoring of the biochemical oxygen demand (BOD) of the treated effluent is essential. However, the conventional 5-day BOD (BOD_5_) test is laborious, sometimes poorly reproducible and inaccurate, particularly at low concentrations of organic compounds [1]. Moreover, membrane fouling of the dissolved oxygen probe can become a serious problem with wastewater, and frequent maintenance is therefore needed to maintain high sensitivity [2]. These issues supported the development of microbial biosensors for BOD measurement, based on respirometry [3,4], bioluminescence [5,6], bioreactor/chemostat technology [7,8], and structure-based biodegradation estimation [9]. Biosensors provide the additional benefit of automation; however, they usually make use of single microbial strains, which may not be able to oxidize a broad range of substrates. Consequently, dissolved oxygen consumption in such sensors may not be directly proportional to the total concentration of biodegradable organics present in solution [10]. In addition, the intrinsic limitation of oxygen solubility in aqueous solutions, the short-term stability of probes, and the lysis of immobilized microbial strains challenge the viability of such sensors [11,12].

In recent years, microbial fuel cells (MFCs) have been examined as an alternative BOD sensing device [1,2,13,14,15,16,17]. MFC BOD sensors are quick and portable, and contain a mixed population of microorganisms (by using environmental inoculum from a wastewater reclamation plant in Singapore) able to oxidize a wide range of substrates [1,18]. Yet the design and operation conditions as reported in the literature are suboptimal, consisting of two-chambered MFC systems [13,14] functioning in batch mode [15,16,17]. For the operator of a wastewater treatment plant to continuously monitor the evolution of BOD in effluent samples, a continuous “online” system is needed. Furthermore, the use of single-chambered air cathode MFCs should be preferred, as these are easier and cheaper to operate than their two-chambered counterparts, mostly because the cathode can be directly exposed to ambient air as a free and sustainable source of oxygen.

Another area of improvement for MFC sensors is the choice of an affordable and sustainable cathode catalyst. So far, most sensors reported in the literature have made use of expensive platinum [19,20,21]. However, considering the high cost and low durability of platinum due to corrosion and degradation of catalytic activity over time [22], alternative metal catalysts such as indium/tin [23], cobalt [24], titanium [25], tungsten [26], or manganese [27] should be sought. In our previous work, we explored the efficacy of manganese oxide (MnO_2_) as a catalyst for the oxygen reduction reaction [27]. Specifically, three types of MnO_2_ catalysts (i.e., α-, β-, and γ-MnO_2_), based on different preparation methods and crystalline structure, were incorporated individually into three air-cathode MFCs. The performance of the MFCs were characterized and investigated by cyclic voltammetry in neutral medium. The performance of the MFCs based on their electrical performance showed the superior catalytic activity of β- and γ-MnO_2_ over α-MnO_2_; yet, the efficacy and long-term stability of such catalyst in a sensor device remains to be assessed.

In this study, a compact single-chambered MFC, with an air cathode and MnO_2_ as a cathode catalyst, was tested as a BOD sensor in a continuous mode. With the aim of implementing this MFC BOD sensor in wastewater treatment plants, the performance stability of the sensor must be evaluated over a long time period in order, for example, to address the repeatability of results and the need to re-calibrate the sensor over time. Therefore, unlike previous studies, the performance of the BOD sensor was evaluated over 1.5 years in this study using both a synthetic substrate and real domestic wastewater (DWW). The performance of the MFC BOD sensor was assessed holistically in terms of the linear relationship between signal output and organic concentration, sensor stability, and the compliance of predicted BOD values using the MFC biosensor with BOD_5_, obtained according to standard methods [28].

## 2. Results

### 2.1. Optimization of Hydraulic Retention Time (HRT)

**Figure 1:**
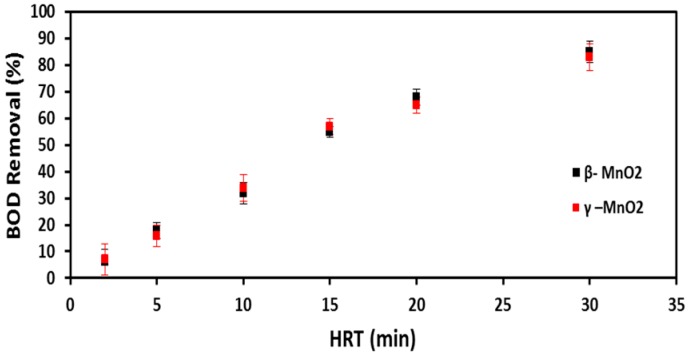
Optimization of hydraulic retention time (HRT) for the β-and γ-MnO_2_-type microbial fuel cell (MFC) biochemical oxygen demand (BOD) sensors by measuring the BOD_5_ removal efficiency at different HRTs.

A good sensor should measure “true” BOD_5_ concentrations, provide a strong signal (in this case, voltage output), and have a quick response time. For this purpose, the HRT of the MFC-based BOD sensor was optimized. A sodium acetate solution at a fixed BOD_5_ concentration of 386 ± 10 mg/L was used to avoid the variability of DWW and study the reproducibility of the MFC-based BOD sensor. Figure 1 shows that the BOD_5_ removal rate decreased with decreasing HRT from 30 to 2 min. It was observed that an HRT of 30 min removed 85% of BOD_5_ in the influent feed, while an HRT of 2 min decreased the BOD_5_ removal efficiency down to 6% for both β- and γ-MnO_2_-type BOD sensors. Only 6% of BOD_5_ removal efficiency at the HRT of 2 min ensured that the MFC BOD sensor would measure BOD values substantially similar to the BOD_5_ concentration present in the influent feed. The response time of the BOD sensors was calculated at the HRT of 2 min by alternating the phases of feeding and starvation periods and monitoring the time taken for the voltage output to reach a maximum stable voltage value. The shortest response time of the MFC BOD sensors was measured in the range of 2–3 h at a 2 min HRT for both β- and γ-MnO_2_-type BOD biosensors.

### 2.2. Correlation between BOD_5_ and Voltage Output

#### 2.2.1. Calibration with Sodium Acetate Solution

**Figure 2:**
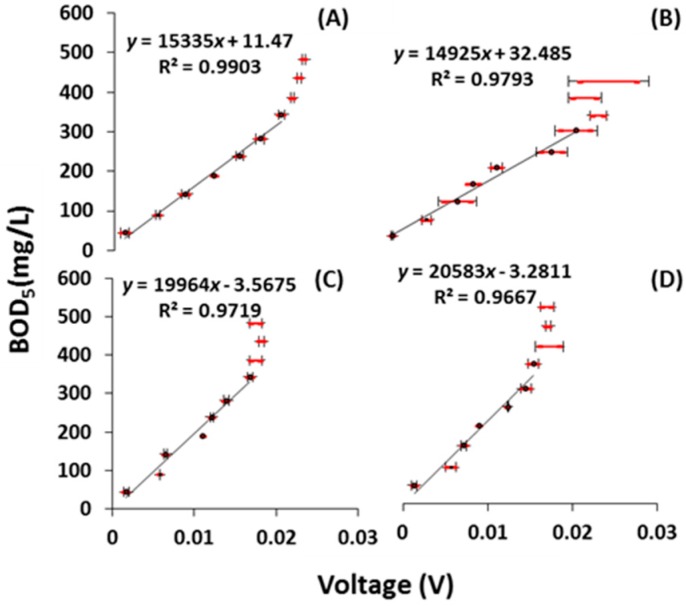
Dynamic response of the MFC BOD sensors against organic concentration (BOD_5_) in sodium acetate solution at different time intervals. (**A**,**B**) show a linear relationship between maximum steady-state voltage generated and BOD_5_ of the β- and γ-MnO_2_-type MFC BOD biosensors, respectively, after 3 months; (**C**,**D**) demonstrate the same type of linear relationship after 1 year. Standard deviations in voltages are indicated in red color.

After three months of operation, the sensors were calibrated with the sodium acetate solution at different BOD_5_ concentrations ranging from 44 to 483 mg/L (Figure 2A,B). A linear relationship was obtained between the BOD_5_ and the maximum steady-state voltage as long as the BOD_5_ remained in the range of 44–343 mg/L. The *R*^2^ value was high at 0.99 and 0.97 for the β- and γ-MnO_2_, respectively. The reproducibility of the sensor response was found to be better with β-MnO_2_ (standard deviation of 0.05%) than with γ-MnO_2_ (standard deviation of 0.22%). As shown in Table 1, the *F*-values were 511 and 236 for the β- and γ-MnO_2_ type MFC BOD biosensor, respectively, implying that the linear model was significant. Additionally, the value of “Prob > *F*” (probability that null hypothesis is correct) remained below 0.05, indicating that the model terms were significant at a 95% level of confidence. In both types of sensor, a good relationship was obtained between the voltage output and the BOD_5_, indicating reliable performance and calibration. 

The performance of the sensors was determined again under the same conditions after one year of operation of the MFC BOD sensor (Figure 2C,D). Again, a good linear relationship was obtained for a BOD range of 44–343 mg/L with an *R*^2^ value of 0.97 (*F*-value of 173) and an *R*^2^ value of 0.96 (*F*-value of 145) for the β- and γ-MnO_2_-type BOD sensors, respectively. All the experiments were conducted in duplicate, and the standard deviation equaled 0.075% and 0.16%, for the β- and γ-MnO_2_-type MFC BOD sensors, respectively. 

#### 2.2.2. Calibration with Domestic Wastewater (DWW)

**Figure 3:**
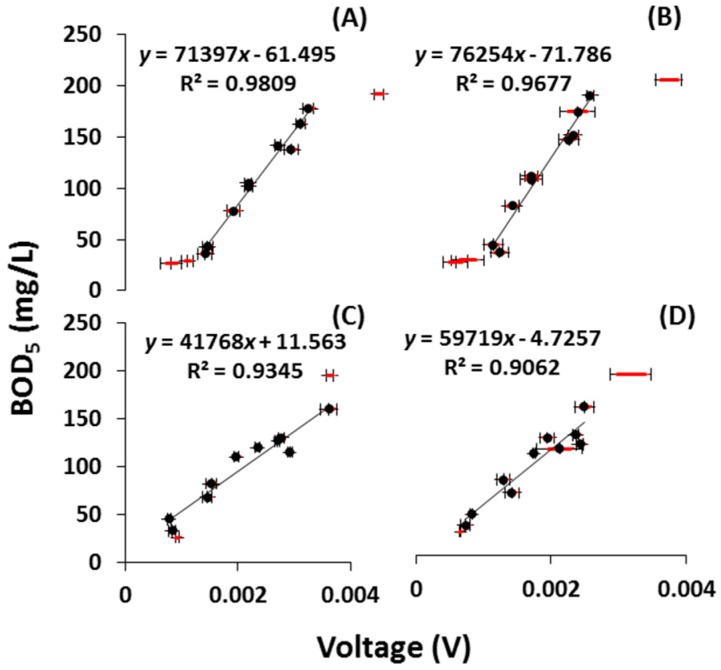
Dynamic response of MFC BOD sensors against organic concentration (BOD_5_) in DWW. (**A**,**B**) show a linear relationship between the independent variable (maximum voltage) and dependent variable (BOD_5_) of the β- and γ-MnO_2_-type MFC BOD sensors, respectively, after 6 months; (**C**,**D**) demonstrate the same type of relationship after 1.5 years. Standard deviations in voltages are indicated in red color.

For practical applications, the MFC BOD sensors must be able to work with DWW; therefore, this was tested both with β- and γ-MnO_2_ after six months of operation. In those conditions, a linear relationship between the voltage output and the BOD_5_ concentration was again obtained, confirming the results obtained with synthetic substrate. Figure 3A,B show a linear relationship between the BOD_5_ (ranging from 36 to 178 mg/L) and the maximum steady-state voltage with *R*^2^ values of 0.98 and 0.96, and *F*-values of 360 and 209, with β- and γ-MnO_2_, respectively. Additionally, the very low value of model parameter, “Prob > *F*” indicated that the model was statistically significant. To complete a comprehensive examination of the long-term stability of the MFC BOD sensors, they were finally tested again with DWW after 1.5 years (Figure 3C,D). When measuring their performance with respect to BOD_5_, which ranged from 33 to 160 mg/L, the regression coefficient, *R*^2^, was 0.93 and 0.90, and the *F*-value was 114 and 77, for the β- and γ-MnO_2_-type MFC BOD sensors, respectively. Table 1 summarizes the overall performance of both types of MFC BOD sensors and shows that the performances in terms of regression coefficient and *F*-value were slightly decreased after 1.5 years. Nonetheless, the MFC BOD sensors were still able to measure the BOD of water samples with good accuracy, confirming the longevity of MFC BOD sensors. 

### 2.3. Compliance of Predicted BOD Values (BOD_p_) with BOD_5_

**Figure 4:**
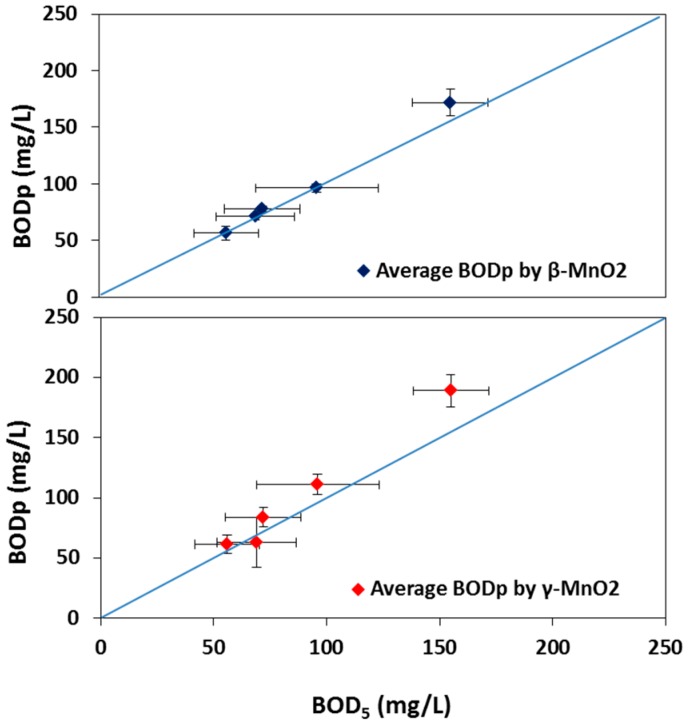
Variation between BOD_p_ by the β- and γ-MnO_2_-type MFC BOD sensors and the BOD_5_ measured with the conventional 5-day BOD method.

The compliance of the BOD values as predicted by the MFC sensors (BOD_p_) with BOD_5_ was determined using five random samples of DWW after 1.5 years of operation, using the calibration curves obtained at that time. Figure 4 plots the BOD_p_ values measured with β- and γ-MnO_2_ MFC BOD sensors against the BOD_5_ for all five samples. The BOD_5_ of the five samples ranged from 56 to 155 mg/L, with a standard deviation comprised between 14%–27%. For both types of catalysts, a good correlation was obtained between BOD_5_ and BOD_p_; yet the standard deviation obtained with β-MnO_2_ (3%–12%) was lower than that of γ-MnO_2_ (8–21%). Nevertheless, these standard deviations remained lower than that of BOD_5_, therefore showing the improved reliability of the sensors over the conventional BOD_5_ method, especially when β-MnO_2_ was used as the catalyst.

## 3. Discussion

### 3.1. Impact of Operating Parameters

For achieving reliable performance of any BOD sensor, the “true” concentration of BOD_5_ must be measured; the concentration of BOD_5_ is almost the same in both the influent and effluent wastewater feed. In other words, a lower BOD_5_ removal rate indicates the measurement of the “true” influent BOD_5_ concentration, which is the desired BOD_5_ measurement. The observation of a decreasing BOD_5_ removal rate with a decreasing HRT illustrates how a lower HRT may help to measure nearly an equal organic concentration, which is present in the influent feed. Moreover, a lower HRT increases the electrochemical activity of the biofilm by increasing mass transfer rate and subsequently helps in decreasing the response time of the MFC BOD sensor. These results are in accordance with the findings of Di Lorenzo et al. [19], who reported that the dynamic response of MFC sensors became faster at lower HRTs (experiments were conducted at HRTs ranging from 89.3 to 417.0 min).

### 3.2. Impact of Catalysts

In the previous study, β-MnO_2_ was reported as yielding the best performance, as assessed by cyclic voltammetry and confirmed by actual MFC experiments [27]. Nevertheless, the performance of γ-MnO_2_ was good enough to be assessed for its efficacy as an MFC BOD sensor. The present study confirms the superiority of β-MnO_2_ MFC sensors over γ-MnO_2_ sensors in terms of sensitivity and stability. This can be explained by different crystallographic structures of β- and γ-MnO_2_ catalysts. Indeed, β-MnO_2_ presents a homogeneous nano-rod structure, while γ-MnO_2_ forms fine needles, dispersed in clusters around carbon nanotubes [27]. The improved structure of the former is likely to allow for better adsorption of O_2_ on its surface and achieve overall higher catalytic activity. In our MFC BOD sensor, this translated into a stronger and more stable signal. This is in accordance with other studies that have correlated the higher catalytic activity of β-MnO_2_ with its higher average oxidation state, as compared to γ-MnO_2_ [29].

### 3.3. Impact of the Substrate

One of the main goals of this study is to provide the operators of wastewater treatment plants with an efficient and sustainable BOD sensor; for this purpose, it is essential to assess the sensor performance not only with synthetic feed but also with real DWW. It is a well-known fact that simple substrates like acetate are more readily assimilable at the anode of an MFC than complex effluents, leading to higher coulombic recoveries [30]. Additionally, a complex substrate such as DWW helps more in the development of electrochemically active biofilms; meanwhile, as an easily consumable feed, a simple substrate improves voltage generation [31]. Indeed, in an MFC BOD sensor, lower assimilable rates of complex organics in DWW lead to slightly reduced sensitivity; nevertheless, a good linear relationship was established with DWW too, at a BOD_5_ ranging between 33 and 172 mg/L, even after 1.5 years, making the MFC BOD sensor applicable for practical use.

### 3.4. Long-Term Stability

Over a long time period of 1.5 years, the voltage generated by both the β- and γ-MnO_2_ MFC BOD sensors eventually decreased, probably due to changes in biofilm composition, bio-fouling on the proton exchange membrane [32], and decreased catalyst activity [33]. Kim et al. [18] reported that their MFC BOD sensor remained stable for five years, but provided little evidence to support their claim. In particular, re-calibration was not addressed. In this study, we noticed that re-calibration is one of the critical factors that affected the performance of the MFC BOD sensor and that it was definitely needed after a year; however, the exact frequency of re-calibration should be evaluated in future works in order to maintain a good proportionality between BOD_5_ and the recorded voltage at all times.

### 3.5. BOD_p_ vs. BOD_5_

The β-MnO_2_-type sensor showed a close compliance of BOD_p_ with BOD_5_, while both MFC BOD sensors exhibited low standard deviation in the repetition of measuring BOD_p_. Results indicated that, for higher BOD values (>150 mg/L), the MFC BOD sensors had a tendency to slightly overestimate the BOD. Yet, the compliance was extremely good in a lower BOD range (between 50 and 100 mg/L), which corresponds to the typical BOD of treated effluents from wastewater treatment plants. This, along with good accuracy, result reproducibility, a fast response time, and an ability to measure BOD, continuously emphasizes the suitability of the MFC BOD sensor developed in this study for application in wastewater treatment plants.

## 4. Materials and Methods

### 4.1. MFC Construction and Operation

**Figure 5:**
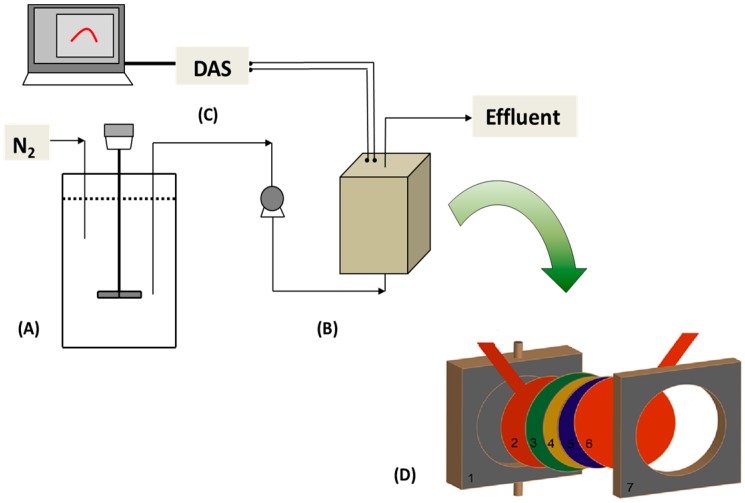
Schematic diagram of the MFC BOD sensor used in this study. (**A**) Influent tank; (**B**) Microbial fuel cell; (**C**) Computer and data acquisition system (DAS); (**D**) Details of MFC configuration—1: MFC body; 2: anode carbon cloth with current collector; 3: Nafion membrane; 4: catalyst layer; 5: carbon base layer; 6: cathode carbon cloth with current collector; 7: cathode frame.

The MFCs used in this study were designed and constructed as acrylic single-chambered air-cathode cylindrical reactors, with a diameter of 6 cm and a width of 1 cm (i.e., a total volume of 28.3 mL) (Figure 5). MnO_2_ nanoparticles were mixed with carbon nanotubes as the conductive material and polyvinylidene fluoride as the binder. Both the anode and cathode consisted of carbon cloth (E-Tek, Woburn, MA, USA), with the cathode coated on one side with MnO_2_ ink (either β or γ) at a catalyst loading of 3 mg·cm^−2^ following the procedure of Lu et al. [27]. The cathode was then hot-pressed together with Nafion, a proton exchange membrane (PEM). All MFCs were duplicated.

The MFCs were inoculated and operated initially with real DWW collected from the primary clarifier of a wastewater treatment plant in Singapore. DWW was used as-is, except for preliminary screening, used with a 0.45 mm pore-sized filter cloth to remove big particles from accumulating inside the MFCs. DWW had a COD of 355.0 ± 75.0 mg/L, total suspended solids of 218.5 ± 105.0 mg/L, total dissolved solids of 110.2 ± 62.3 mg/L, a pH of 7. ± 0.4, and a conductivity of 0.855 ± 0.085 ms/cm. In some instances, a sodium acetate solution was used, prepared according to methodology described by Lefebvre et al. [24]. Influent feeding was done using peristaltic pumps (Masterflex 07523-70, Spectra-Teknik Pte Ltd., Singapore) in an upflow mode. A 10 Ω resistor was connected externally to each MFC, following recommendations by Di Lorenzo et al. [19] that low external resistance can shorten the response time, and the voltage over the resistor was recorded at fixed intervals of 1 h using a digital multimeter connected to a computer via a data acquisition system (M3500A, Array Electronic, Taipei, Taiwan).

### 4.2. Synthesis of MnO_2_ Catalysts

The β-MnO_2_ catalyst was prepared by dissolving MnSO_4_·H_2_O (1.6 mmol) and (NH4)_2_S_2_O_8_ (1.6 mmol) in 20 mL of water at ambient temperature (~25 °C) to form a homogeneous solution, which was then transferred to a Teflon-lined stainless steel autoclave, sealed, and maintained at 125 °C for 14 h. After the solution was cooled down to ambient temperature, the resulting dark-gray solid product was filtered, washed, and dried. γ-MnO_2_ was prepared by dissolving MnSO_4_·H_2_O (16 mmol) and (NH_4_)_2_S_2_O_8_ (16 mmol) in 200 mL of water. After that, the mixture was heated up to 9 °C in an oil bath and maintained for 24 h with magnetic stirring. The resulting black precipitate was filtered, washed, and dried [27].

### 4.3. Calibration Procedure

The BOD sensor was calibrated by plotting the steady-state voltage (the signal) against BOD_5_ in a linear relationship. Since a fixed external load of 10 Ω was used in all experiments, the relationship between current and voltage was proportional and followed Ohm’s law. For calibration with the sodium acetate solution, the MFC BOD sensors were fed with a specific BOD_5_ until the maximum steady voltage was generated and recorded. Between two sets of experiments, a period of starvation allowed the voltage to drop to 1.5 ± 0.5 mV. The MFC BOD sensor was fed with acetate solutions with different BOD_5_ concentrations, and each concentration was tested twice to ensure reproducibility. For calibration with DWW, a similar methodology was used with several dilutions of feed prepared using deionized water.

### 4.4. Analyzes

BOD_5_ and COD tests were performed in accordance with standard methods [28], using the 5-day dilution method and the closed reflux titrimetric method, respectively. The samples were filtered through glass microfiber filters (0.45-μm pore-sized, Whatman, Irvine, UK) prior to analysis. The analysis of variance (ANOVA) was used as a statistical analysis tool to examine and interpret the linear model equations obtained during calibration. ANOVA is a simple regression analysis that uses the Fisher’s *F*-test, and its associated probability to assess the robustness of linear models and overall model significance. A “Prob > *F*” less than 0.05 and a large *F*-value indicate that the model terms are significant. 

## 5. Conclusions

This study is first to use an MnO_2_ catalyst (in place of an expensive Pt catalyst) for MFCs applied as online BOD sensors. A good linear relationship between the voltage and BOD_5_ in both a sodium acetate solution and DWW, a stable performance in the long term (more than 1.5 years of operation), and close compliance between BOD_p_ and BOD_5_ demonstrated the potential of MnO_2_-MFC BOD sensors in substituting the conventional BOD_5_ methods, which takes a longer analytical time of five days and suffers from substantial variability.

## Figures and Tables

**Table 1:** Overall performance shown by the β- and γ-MnO_2_-type microbial fuel cell (MFC) biochemical oxygen demand (BOD) sensors.

Type of Sensor	Time Period	Type of Wastewater	Variables for Polynomial Regression	Range of Organic Concentration (BOD_5_) Measured (ppm)	*R* Square	*F*-Value	Prob > *F*
β-MnO_2_	3 months	AWW	Voltage vs. BOD	44–343	0.990	511	3.14 × 10^−6^
β-MnO_2_	6 months	DWW	Voltage vs. BOD	36–178	0.980	360	2.281 × 10^−7^
β-MnO_2_	1 year	AWW	Voltage vs. BOD	44–343	0.971	173	4.55 × 10^−5^
β-MnO_2_	1.5 years	DWW	Voltage vs. BOD	33–160	0.934	114	5.18 × 10^−6^
γ-MnO_2_	3 months	AWW	Voltage vs. BOD	44–343	0.979	236	2.12 × 10^−5^
γ-MnO_2_	6 months	DWW	Voltage vs. BOD	36–178	0.967	209	1.79 × 10^−6^
γ-MnO_2_	1 year	AWW	Voltage vs. BOD	44–343	0.966	145	6.93 × 10^−5^
γ-MnO_2_	1.5 years	DWW	Voltage vs. BOD	33–160	0.906	77	2.20 × 10^−5^
